# Detection of Volatile Metabolites of Garlic in Human Breast Milk

**DOI:** 10.3390/metabo6020018

**Published:** 2016-06-06

**Authors:** Laura Scheffler, Yvonne Sauermann, Gina Zeh, Katharina Hauf, Anja Heinlein, Constanze Sharapa, Andrea Buettner

**Affiliations:** 1 Department of Chemistry and Pharmacy, Emil Fischer Center, Friedrich-Alexander-Universität Erlangen-Nürnberg (FAU), Henkestr. 9, Erlangen 91054, Germany; laura.scheffler@fau.de (L.S.); yvonne.sauermann@fau.de (Y.S.); gina.zeh@fau.de (G.Z.); Katharina.hauf@fau.de (K.H.); anja.heinlein@fau.de (A.H.); constanze.sharapa@fau.de (C.S.); 2 Fraunhofer Institute for Process Engineering and Packaging IVV, Giggenhauser Str. 35, Freising 85354, Germany

**Keywords:** garlic, human milk, gas-chromatography mass-spectrometry, allyl methyl sulfide, allyl methyl sulfoxide, allyl methyl sulfone

## Abstract

The odor of human breast milk after ingestion of raw garlic at food-relevant concentrations by breastfeeding mothers was investigated for the first time chemo-analytically using gas chromatography−mass spectrometry/olfactometry (GC-MS/O), as well as sensorially using a trained human sensory panel. Sensory evaluation revealed a clear garlic/cabbage-like odor that appeared in breast milk about 2.5 h after consumption of garlic. GC-MS/O analyses confirmed the occurrence of garlic-derived metabolites in breast milk, namely allyl methyl sulfide (AMS), allyl methyl sulfoxide (AMSO) and allyl methyl sulfone (AMSO_2_). Of these, only AMS had a garlic-like odor whereas the other two metabolites were odorless. This demonstrates that the odor change in human milk is not related to a direct transfer of garlic odorants, as is currently believed, but rather derives from a single metabolite. The formation of these metabolites is not fully understood, but AMSO and AMSO_2_ are most likely formed by the oxidation of AMS in the human body. The excretion rates of these metabolites into breast milk were strongly time-dependent with large inter-individual differences.

## 1. Introduction

Human milk is commonly the primary and often sole food source of a newborn during the early stages of perinatal life. The specific composition of human milk adapts during an infant’s development to satisfy its nutritional requirements; it is thus commonly recommended that mothers breastfeed their children at least during the first six months after birth [1]. Although the nutritional adaptation of the milk proceeds more or less irrespective of the maternal diet, there is evidence that the latter can have an influence on its odor. Several studies report that the suckling behavior and later dietary habits of infants may be influenced by the mothers’ choice of diet during pregnancy and nursing. For example, infants whose mothers consumed carrot during pregnancy and breastfeeding periods accepted carrot-flavored cereals more readily than infants who were exposed to this flavor for the first time [2]. Likewise, after ingestion of garlic [3], alcohol [4], and vanilla flavor [5], the infants’ behavior was altered, displaying intensified suckling and longer breast attachment. These observations have led to the assumption that potential flavor changes of human milk can be detected by the infant during breastfeeding. Other studies have made similar observations of odor changes in human milk after mothers had ingested garlic [3], beer [6] or carrot juice [2], although it was only reported that mothers observed a difference; the exact nature of these sensorial changes was not characterized by an expert panel, and the underlying molecular processes remained unclear. On the other hand, other studies found no evidence of the transfer of odorous constituents into human milk from foods such as herbal tea [7] or fish oil [8], as determined by sensory evaluations and chemical analysis. In view of this, it is important to note that sensorial changes in human milk readily occur within relatively short periods due to oxidation processes, thereby rendering unclear whether these changes reported by mothers in previous studies relate to such oxidation effects rather than direct odorant transition [9,10,11,12]. Accordingly, an expert sensory rating appears indispensable in terms of elucidating a relationship of human milk aroma changes to specific food interventions.

The molecular and physiological basis of the transfer of odor compounds into human milk is complex and not yet fully understood. Several factors including the milk fat content or the absorption in the gastrointestinal tract may influence the transmission of odorants. Additionally, further metabolism steps, e.g., oxidation of the compounds, may take place prior to their excretion into human milk. Such functionalization has been demonstrated for 1,8-cineole (eucalyptol) with the formation and secretion of a number of derivatives into human milk after oral administration of this substance [13,14]. Other studies have similarly reported the transfer of specific flavor compounds into human milk. Hausner *et al.* [15], for example, demonstrated the transfer of d-carvone, *trans*-anethole and l-menthol to human milk when administering encapsulated odorants. On the other hand, in the same study, 3-methylbutyl acetate was not detected in human milk despite the relatively high dosage, indicating biotransformation of the compound *in vivo*. Recently, Kirsch *et al.* [13] confirmed the transfer of 1,8-cineole to human milk, although in this and the latter study relatively high concentrations of the substances were applied. The rationale in the case of the study by Kirsch *et al.* [13] was to investigate dosages that are common in pharmacological preparations and that are also prescribed to breastfeeding women; in this case this related to capsules containing 100 mg 1,8-cineole. Likewise, Hausner *et al.* [15] used capsules containing 100 mg of the respective compounds to standardize administration for monitoring the subsequent potential odorant transfer into human milk. Nevertheless, the use of such odorant models does not accurately reflect real-life food consumption whereby the complex food matrix of an everyday meal is commonly composed of different constituents such as meat, vegetables and other sides, thus containing an array of aroma constituents. Accordingly, conclusions regarding everyday food consumption are difficult to draw from the insights obtained by studies on model systems.

The potential aroma transfer into human milk is therefore not a conditional effect but must be regarded as a variable process that is dictated by the specific chemical structures as well as quantitative composition of the aromas applied. Obviously, not all aroma compounds that are consumed reach the milk unchanged, but metabolism or general biotransformation may occur. In view of this, it is important to note that thio-containing aromas, such as garlic aroma, have hitherto never been investigated for their potential modification of human milk aroma on a molecular basis. Such analytical investigations present a great challenge due to the typically low concentrations of these substances that exhibit extremely high odor potency.

Based on these considerations, the aim of this study was to determine whether ingestion of garlic influences the overall aroma of human milk; the main consideration hereby was to follow a consumption protocol that accurately reflects real-life garlic consumption. In our study, garlic was chosen based on previous studies, which indicated that garlic alters the aroma profile of human milk [3]. Furthermore, garlic is frequently consumed by humans and has not been reported to induce any side effects in the infants when consumed in normal quantities [16]. In contrast to the study by Mennella *et al.* [3], which investigated human milk after the consumption of capsules containing 1.5 g garlic extract, we aimed at investigating the influence of fresh garlic on human milk when consumed at food-relevant concentrations. In doing so, we aimed at demonstrating if dietary-relevant amounts of garlic have an influence on the aroma of human milk.

The second aim of our study was to identify the molecules which are responsible for the potential change of the odor of human milk and to characterize their relative temporal transfer into the milk. An identification of the underlying molecules present in the milk reveals whether these are the original food constituents (in this case odor constituents of garlic) that are indeed directly transferred into the milk, as is commonly believed, or whether these constituents have undergone metabolism before being excreted into the milk. These findings will broaden our understanding of the physiological processes occurring in the maternal body and how infants are influenced by the maternal diet, both on a sensory but also a molecular basis.

## 2. Results

### 2.1. Determination of Odor Qualities of Reference Compounds

A series of garlic odorant or potential metabolite compounds was freshly synthesized for use as reference compounds; their synthesis was necessary as most were not commercially available (*cf.* 4.2). Moreover, the odor qualities of some reference compounds had to be determined prior to performing the study due to an absence of data on these particular compounds. The compounds evaluated were diallyl sulfoxide (DASO), diallyl disulfone (DASO_2_), allyl methyl sulfoxide (AMSO) and allyl methyl sulfone (AMSO_2_). DASO was described by the panel as garlic-like. DASO_2_ on the other hand was not perceivable at all, even at high concentrations of up to 477.5 µg/mL. AMSO and AMSO_2_ were similarly odorless. The analytical and olfactometric data obtained for all compounds, together with literature reports, are presented in Table 1. Table 1 further indicates if these substances were detectable in breast milk in this study, as will be further detailed in Section 2.3 and Section 2.4.

### 2.2. Aroma Profile Analysis

**Figure 1:**
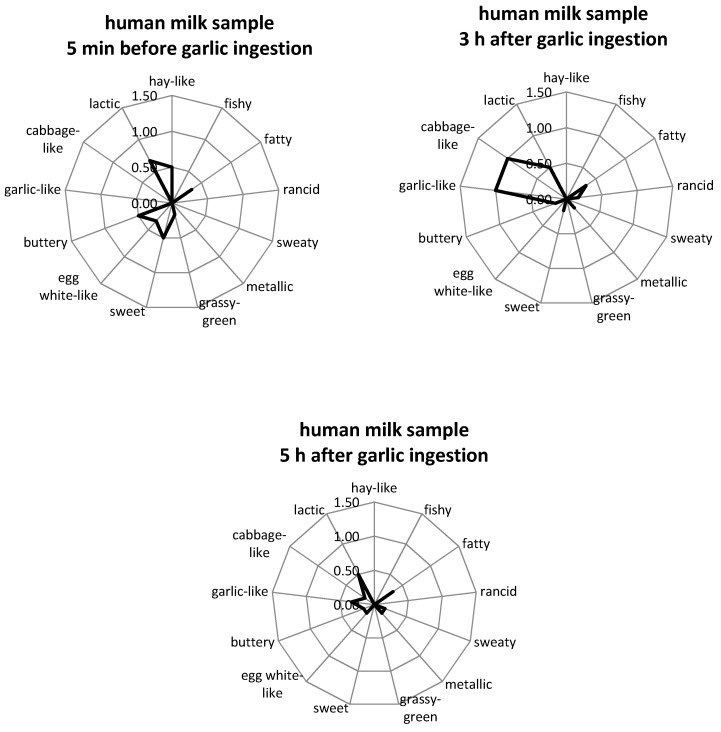
Odor profiles of human milk samples of test person *c*, as a representative example. The samples were collected at different times before and after ingestion of 3 g raw garlic. Panelists were asked to rate the orthonasal perception on a scale from 0 (no perception) to 3 (strong perception). Values are mean ratings of all panelists. Note: The scale is only presented up to the value of 1.5 for better visualization.

Comparative aroma profile analysis (APA) was performed on milk samples taken before and approx. 2–3 and 4–5 h after garlic consumption (depending on the lactation period of the mother) in order to assess possible odor changes of the respective breast milk samples due to the garlic intervention. The following attributes were found to be adequate descriptors: fishy, fatty, metallic, grassy-green, rancid, sweaty, buttery, sweet, hay-like, egg white-like and lactic, plus garlic- and cabbage-like. The attributes were chosen based on those which were used to describe the odor of human milk in other studies [39], as well as on descriptors selected by our sensory panel in preliminary sensory experiments with breast milk obtained after garlic consumption. All odor attributes associated with the typical milk odor were rated as having very low odor intensities and in most cases were not perceivable (0) or just detectable (1), which is in agreement with previous studies [10,39]. In rare cases, single panelists judged some impressions as being intense. Garlic- and cabbage-like attributes were not perceivable in any of the milk samples taken before the mothers consumed garlic. By comparison, the samples collected after garlic consumption were judged by the majority of the panelists as having a slight to average garlic- or cabbage-like odor (intensities 0.5–2). A selection of aroma profiles of human milk samples that were taken over a period of 5 h are shown in Figure 1. All APA results are available in Figure S1 of the supplementary material.

### 2.3. Comparative Aroma Extract Dilution Analysis (cAEDA) of the Milk before and after Garlic Consumption

Comparative aroma extract dilution analysis (cAEDA) [40,41] was performed for the extracts of the milk samples taken before and after garlic consumption to characterize the odor-active compounds which are responsible for the garlic-/cabbage-like odor in the milk samples after garlic consumption. cAEDA showed that only one substance with a garlic-like odor and retention index (RI) < 1000 on the FFAP capillary and RI 715 on the DB-5 capillary was detectable as an additional substance in those samples that were collected after garlic consumption compared to the controls before garlic consumption. This substance was identified as AMS based on the comparison of the odor quality and the respective RIs with reference substances (see Table 1). All remaining substances that were detected are common odor substances in human milk, including several fatty acids and lactones, as reported in previous studies [10,42]. These compounds are therefore not reported here in further detail.

### 2.4. Identification of Garlic-Derived Metabolites in Human Milk

A targeted search of the potential presence of odorless or less potent garlic-derived metabolites in human milk was performed via high-resolution gas chromatography−mass spectrometry (HRGC-MS) and two-dimensional high-resolution gas chromatography (HRGC-GC-MS). Chromatograms recorded for the extracts of the milk samples taken before and after garlic consumption were thereby compared to establish whether additional peaks were present in the latter extracts. The samples were also screened for the presence of substances that had already been reported in literature either in human breath after garlic consumption, in garlic, or that had previously been suspected to be metabolites being formed in the human body after garlic consumption (see Table 1); these screening assays were carried out based on the respective original reference substances using both HRGC-MS and HRGC-GC-MS to ensure highest sensitivity in the detection of these compounds. Use of the latter system enabled the successful separation of AMS from other co-eluting substances, which was not possible via HRGC-MS. This was additionally confirmed by HRGC-O analysis (see above).

**Figure 2:**
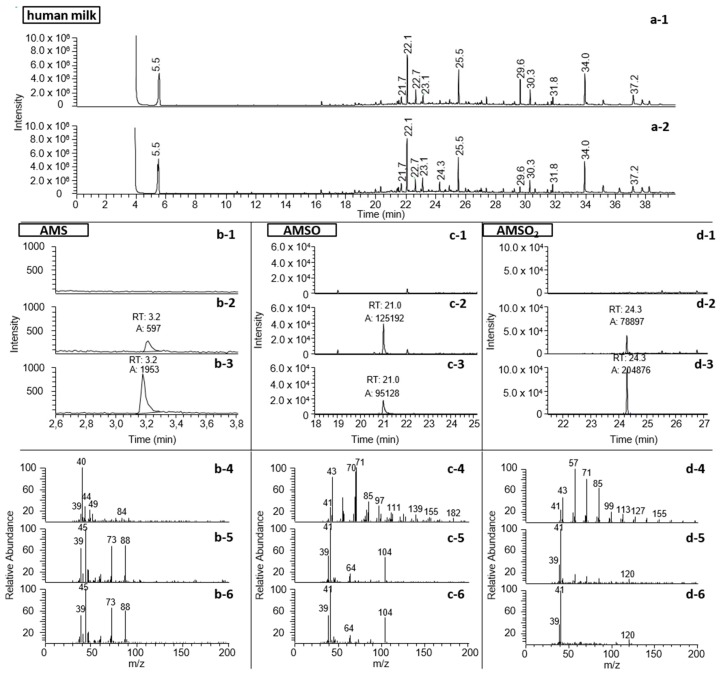
Identification and relative quantification of garlic-derived components in human milk samples of test person *c*. (**a**): Total ion chromatogram of human milk extract from HRGC-MS analysis (FFAP); **b**: AMS, measured with HRGC-GC-MS; *m*/*z* 73 and 88 are extracted for relative quantification; (**c**): AMSO, measured with HRGC-MS (FFAP); *m*/*z* 104 is extracted for relative quantification; (**d**): AMSO_2_, measured with HRGC-MS (FFAP); *m*/*z* 120 is extracted for relative quantification. The human milk extract is shown 5 min before (a-1) and 3 h after (b-1) garlic ingestion. The compounds AMS, AMSO and AMSO_2_ are shown in human milk 5 min before (x-1) and 3 h after (x-2) garlic ingestion as well as the standard compound (x-3), which was used for identification. In x-4 to x-6 the respective mass spectra to x-1 to x-3 are shown. The mass spectra are shown at the time when the standard compound eluted.

Using this approach it was further possible to detect a total of three metabolites, namely AMS, AMSO and AMSO_2_. Example chromatograms are displayed in Figure 2.

**Figure 3:**
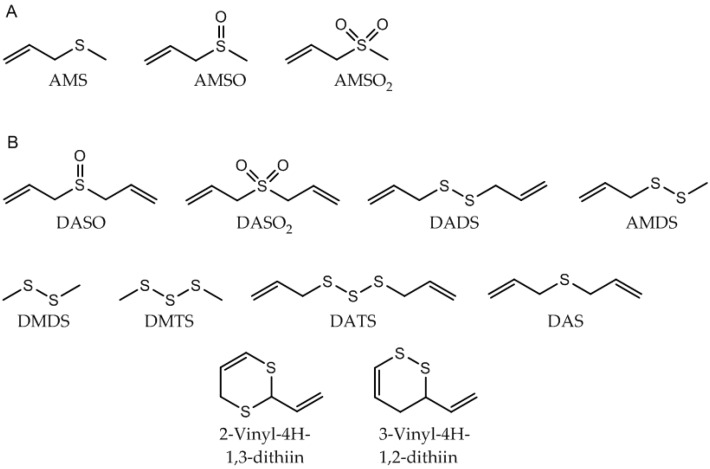
Garlic-associated compounds. (**A**) Detected in human milk after garlic ingestion; (**B**) Not detected in human milk after garlic ingestion. Abbreviations: refer to Table 1.

The presence of AMS in the milk sample prior to garlic ingestion could similarly be excluded (see Figure 2b). The oxidized garlic derivatives AMSO and AMSO_2_ after ingestion of raw garlic were determined in human milk by HRGC-MS (see Figure 2c,d). The other substances which were monitored as possible garlic metabolites were not detectable in human milk either before or after garlic ingestion (see Table 1): these substances were DASO, DASO_2_, DADS, AMDS, DMDS, DMTS, DATS, DAS, 2-vinyl-4*H*-1,3-dithiin and 3-vinyl-4*H*-1,2-dithiin. Accordingly, we can exclude their presence in human milk, at least at any relevant concentrations. The structures of the investigated molecules are shown in Figure 3.

### 2.5. Time Dependency of Appearance of the Garlic-Derived Metabolites in the Human Milk after Consumption of Garlic

**Figure 4:**
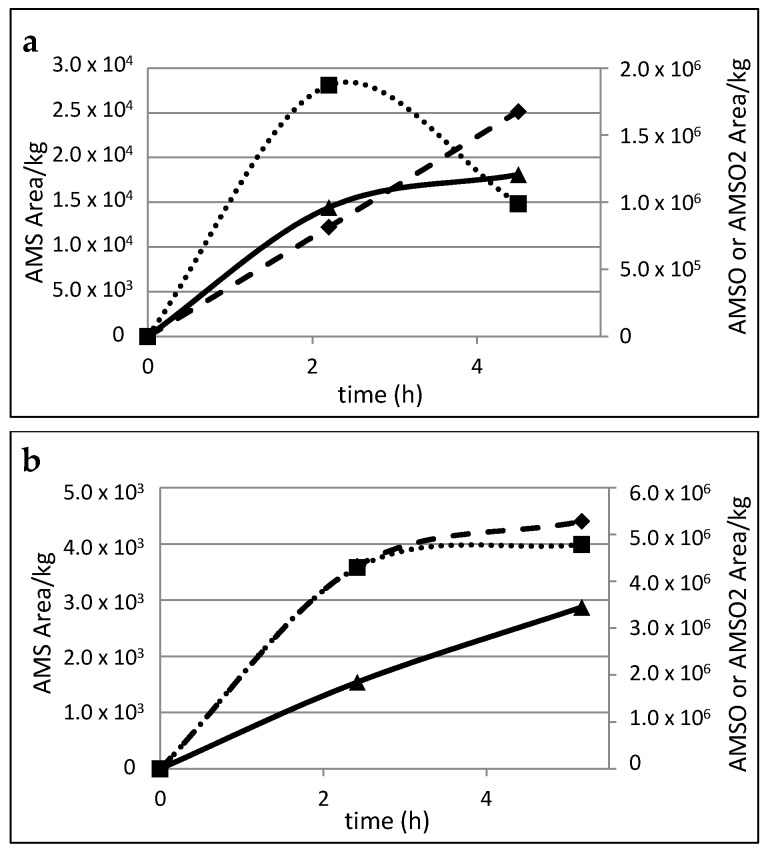

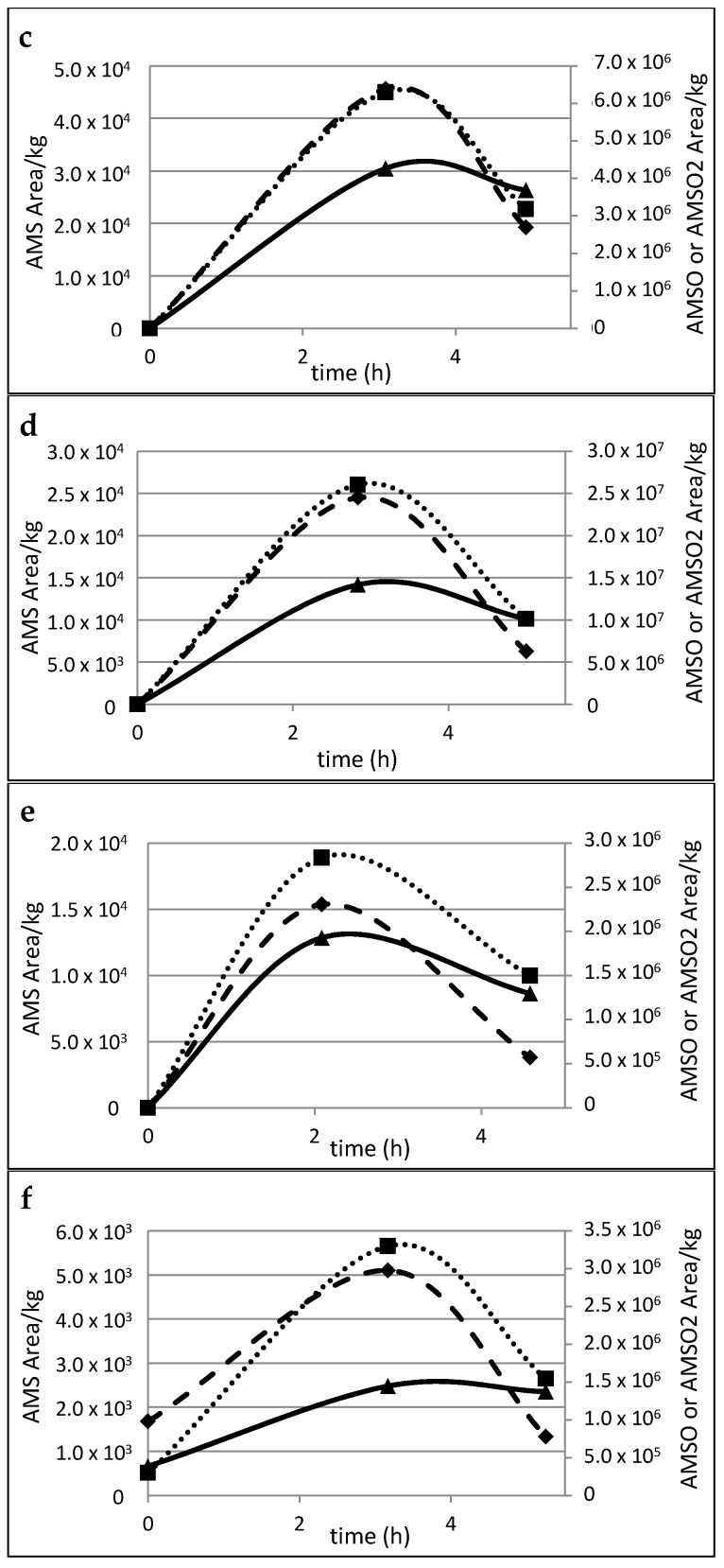
Time-resolved metabolite profiles of AMS, AMSO and AMSO_2_, **a**–**f**: Milk samples taken from six different mothers. AMS (♦), AMSO (■), AMSO_2_ (▲), time 0 h represents the milk sample collected prior to garlic consumption, following times represent milk samples after garlic consumption. Garlic was consumed 5 to 15 min after the first milk sample was given.

The area/kg human milk ratios for the different substances were calculated in order to obtain a relative semi-quantitative estimation of the appearance and the temporal profiles of the identified garlic metabolites in the milk samples. Values were recorded for the milk samples that were taken before and after garlic consumption at different times, and for six individual mothers. The corresponding metabolite profiles for all six mothers are shown in Figure 4.

Although the milk samples were gathered at similar times, the profiles indicate that there are high inter-individual differences in the metabolism and/or excretion rate of the respective garlic constituents. In particular, AMS formation and excretion appeared to be highly variable between different individuals. The relative values obtained for the milk samples from mothers *a* and *b* showed a continuous increase in AMS content over the whole sampling period. Conversely, the highest values of AMS in the milk of mothers *c*, *d*, *e* and *f* were recorded in the second milk sample, which was taken between 2 to 3 h after garlic consumption; thereafter, the content of AMS decreased. In the case of AMSO, the highest value was observed in the second milk sample. Only in the case of mother *b* did the AMSO content increase over the whole sampling time; however, the second increase was noticeably slower than the increase between the first sample (before garlic consumption) and the second sample (2–3 h after garlic consumption). Likewise, AMSO_2_ displayed a continuous increase in the milk of mother *a* as well as in the milk of mother *b* for the respective sampling events. During the collection period, no clear maximum was observed. In contrast to this, however, a decline in AMSO_2_ was observed in the milk samples from mothers *c*, *d*, *e* and *f*, which were taken 4–5 h after garlic consumption.

In the case of the milk of mother *f* it should be noted that traces of garlic metabolites were also found in the milk that was taken prior to garlic consumption, despite the fact that the sensory profile did not reveal any related smell characteristics. This might be due to the fact that the respective mother reported that she consumed some tomato sauce in a restaurant one day prior to the sampling day (see supplementary material Table S6). According to the staff of the restaurant that was consulted regarding this matter, no garlic was used but a broth powder, potentially containing ingredients from onion, leek and garlic. Despite this background effect, we observed a clear increase in the second milk sample taken 3 h after the garlic consumption. In relation to this increase, the amount of AMS in the blank sample was very low, so that it was even not sensorially detectable during GC-O analysis. Interestingly, another mother reported that she was unsure if she potentially consumed some garlic during the wash-out phase with a potato meal that had been prepared for her the day before. However, neither in sensory nor in analytical analysis any indications for garlic smell or metabolite transmission was found in the control milk, so that any relevant impact could be excluded.

## 3. Discussion

### 3.1. Aroma Profile Analysis

The aroma profile analyses showed that the odor of the breast milk changed about 2.5 h after the consumption of raw fresh garlic, which is in agreement with the study by Mennella and Beauchamp [3]. In the latter study [3], the mothers consumed either placebo capsules or capsules containing 1.5 g of garlic extract and the milk samples were collected at hourly intervals, one hour before and up to 3 h after consumption of the capsules. Sensory evaluation of the milk samples showed that samples that were taken 2 h after ingestion of the garlic capsules had higher odor intensities in comparison to the placebo group; these intensities peaked after 2 h and then decreased again. However, the nature of these sensory quality changes was not examined. The present study demonstrates that a clear garlic-/cabbage-like odor appears in human milk samples 2.5 h after consumption of 3 g of raw garlic. The rationale for using 3 g of garlic was that this represents a realistic amount of garlic that may be consumed by a person during a normal diet. On the other hand, no clear difference in the intensities of these descriptors for milk samples taken either 2–3 or 4–5 h after garlic consumption, respectively, were detected. In the case of the milk samples that are shown in Figure 1, the garlic-like attribute was rated with intensities of 1 and 0.33 (mean of all panelists) in the milk samples taken 3 and 5 h after garlic consumption, respectively. For other milk samples, the garlic-like attribute was rated to have the highest in the last milk sample (4–5 h) after garlic-consumption, as can be seen in supplementary material Figure S1. Although this observation might suggest major changes, it nevertheless should be kept in mind that in either case the overall odor intensities were quite low in orthonasal evaluation. Retronasal evaluation (tasting) might have revealed higher sensory intensities, as has been demonstrated before in the case of freeze-stored human milk evaluation [9]. However, retronasal tasting was not carried out by the expert panel in the present study due to work safety considerations. In general, the milk samples were always evaluated directly after expression as our previous work had shown that human milk aroma can undergo quite pronounced sensory changes even after relatively short storage periods [12]. Accordingly, a direct comparison of the samples taken after 2–3 and 4–5 h was not achievable.

### 3.2. Identification of Garlic-Derived Metabolites in Human Milk

In this study, we identified three different garlic-derived metabolites, namely AMS, AMSO and AMSO_2_ after the intake of 3 g raw garlic. Previous studies revealed that food supplements [14] or compounds in high dosages [15] can influence the composition of human milk. During this study we have shown for the first time that dietary-relevant amounts of a food can also have an impact on human milk. In contrast to previous assumptions, however, this study shows that the original aroma constituents are not directly transferred but rather three metabolites, namely AMS, AMSO and AMSO_2_. Accordingly, this is the first report of the excretion of an aroma metabolite into human breast milk in food-relevant conditions. While AMS has previously been found as a metabolite in human breath and in urine after garlic consumption [23,24,25,26], this is the first report of AMS appearance as an odorous garlic-derived metabolite in human milk. Likewise, AMSO and AMSO_2_ were detected here for the first time in human milk after garlic consumption. Both substances are odorless, which explains why neither AMSO nor AMSO_2_ were detected in cAEDA. It is interesting to note that neither of these substances has been found previously in any human bodily fluids or breath after garlic consumption. However, their formation related to garlic consumption has been reported in rat, where both substances were detected in stomach, liver, plasma and urine after administration of diallyl disulfide (DADS) [32]. DADS is a decomposition product of allicin that is formed through the interaction of alliin with allinase after disruption of the cell structure of garlic. It represents 40% to 60% of the essential oil of garlic [32].

Besides providing proof of the appearance of AMS, AMSO and AMSO_2_ in milk after garlic ingestion, we could further exclude the presence of these substances in the milk prior to garlic consumption, with the sole exception of the milk of mother *f*. In this case we observed traces of garlic metabolites that likely relate to prior dietary exposure (see above). This case demonstrates that a wash-out time of at least 24 h prior to the testing time should be adhered to for investigation of such odorant and metabolite transmission effects. In addition, we could further exclude the appearance of a series of other garlic-derived substances in human milk after garlic ingestion, at least for the samples investigated within this study. Future studies are required to explain this preferential appearance of AMS, AMSO and AMSO_2_, which might be related to diverse factors such as (in)stability in the gastro-intestinal tract, biodegradation and bioformation, resorption as well as excretion processes, and the respective quantities of the compounds and their precursors.

### 3.3. Metabolism of Garlic

We observed the excretion of different garlic-derived metabolites over a period of up to 5.2 h. On a semi-quantitative basis, we further observed different excretion profiles with differences in the relative maximum intensities of the monitored metabolites in milk samples from the individual mothers. Temporal differences might relate to variations in milk sampling times, owing to the necessity to adhere to lactation intervals. The metabolism rates might also be influenced by the age of the infant, since the composition of human milk changes according to the lactation period. There are several more potentially influencing factors that might impact the composition of human milk, such as the age or physiological status of the mother, e.g., body mass index [43]. Potential links of these differences with individual metabolism rates or different contents of metabolite precursors in the garlic bulb are thus not discernable from this data. This would require further investigation to first elaborate their potential precursors and to quantify these in relation to the monitored derivatives, not only in breast milk but also taking into consideration other excretion pathways such as urine and breath.

Nevertheless, the main aim of this study was to elucidate the chemical nature of substances and metabolites of garlic in breast milk. In view of this it is important to discuss how the typical garlic-odor of a garlic bulb arises. The release of the garlic smell is caused by the interaction of the enzyme alliinase and the odorless, non-proteinogenic amino acid alliin (*S*-allyl-l-cysteine sulfoxide). These two compounds are located in different cell compartments that only come into contact with each other from cutting or disrupting the cell structure, e.g., by chewing. This then leads to the production of dehydroalanine and allyl sulfenic acid. In a further reaction, two molecules of allyl sulfenic acid may condensate to form allicin [44]. Allicin itself is not stable and can degrade to secondary substances such as ajoenes, vinyldithiins and different sulfides [45]. The exact nature of which metabolites are formed from which precursors, or if and who the different pathways are linked with each other is not presently known.

**Figure 5:**
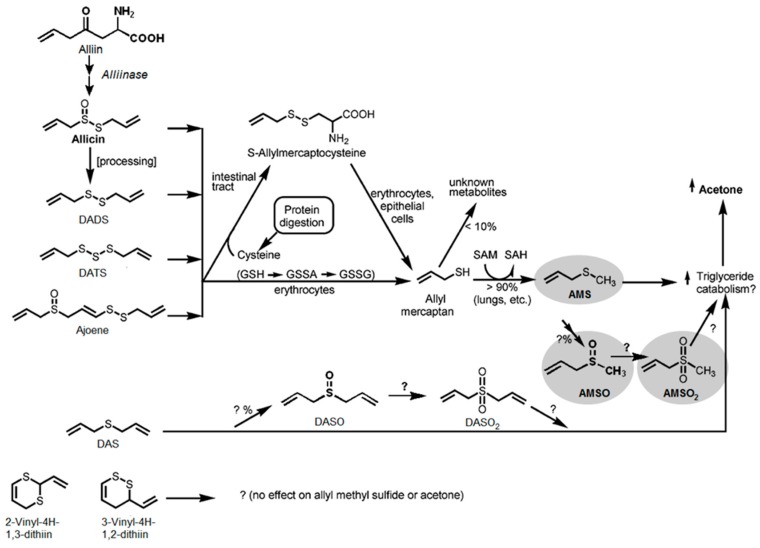
Metabolic fate and metabolic effect of allicin and allicin transformation compounds. GSH: glutathione (γ-Glu-Cys Gly); GSSA: S-allylmercaptoglutathione (γ-Glu-Cys-(S-allyl)-Gly); GSSG: oxidized glutathione, SAM: S-adenosylmethionine; and SAH: S-adenosylhomocysteine. Grey areas mark the components which were found in the present study as human milk metabolites after garlic ingestion. Adapted with permission from Lawson, L.D.; Wang, Z.J. Allicin and allicin-derived garlic compounds increase breath acetone through allyl methyl sulfide: Use in measuring allicin bloavailability. *J. Agric. Food Chem.* **2005**, *53*, 1974–1983 [26]. Copyright (2005) American Chemical Society.

Regarding the metabolism of garlic constituents in the human body, the different pathways are likewise not fully understood. A series of studies addressed this issue, primarily in animal studies involving rats, or in tissue model studies. However, only a few investigations were carried out regarding the metabolism of garlic constituents in human subjects. Lawson and Wang [26] investigated the effect of garlic and garlic-derived compounds on breath composition. Specifically, the authors recorded the acetone and AMS levels in breath after consumption of allicin, allicin-derived compounds (DATS, DADS, DAS, ajoene, distilled garlic oil), allicin metabolites (allyl mercaptan, AMS) and S-allyl cysteine. Administration of these compounds resulted in an increase in acetone and AMS in breath in each case, with the sole exception of DAS and S-allyl cysteine; DAS therefore had no influence on the AMS level but still led to an increase in acetone. Both compounds are the only ones lacking a dithioallyl group. Accordingly, Lawson and Wang [26] concluded that a dithioallyl group is a necessary requirement for the respective precursor substances in the formation of AMS. They also proposed allyl mercaptan as a precursor of AMS. However, as they could show that transformation of allicin to AMS is extremely fast, they did not expect any accumulation of allyl mercaptan under physiological conditions. These observations are in good agreement with our findings as only AMS and its metabolism products AMSO and AMSO_2_ were detected. There were no further compounds detectable that might be considered as precursors of AMS. Apart from that, Lawson and Wang [26] observed that AMS was exhaled sooner than acetone, which indicated that AMS or a metabolite of AMS might be responsible for the increase in breath acetone levels after garlic consumption. Referring to the work of Germain *et al.* [32], the authors further suggested AMSO and AMSO_2_ to be additional metabolites of AMS (see Figure 5), but did not confirm this assumption by experimental data. The present study may be regarded as further support for the proposed formation and occurrence of AMSO and AMSO_2_ as metabolites of AMS in humans. In view of this, it is interesting to note that Lawson and Wang [26] also suggested that DAS is metabolized differently than AMS, since after consumption it is only measureable in trace amounts, but still has an impact on the acetone level in breath. Based on experiments performed by Brady *et al.* [46] and Jin and Baillie [47], they proposed DASO_2_ as a metabolite of DAS (see Figure 5). The results of our study cannot support this assumption, since neither DASO_2_ nor its supposed precursor DASO was present in measurable amounts.

Other studies on garlic breath confirmed the assumption that AMS is a garlic-derived metabolite; they all confirmed exhalation of AMS after ingestion of garlic [17,18,19,20,21,22,23,24,25].

Nevertheless, deeper insights into metabolism of garlic constituents may be obtained from animal and tissue studies. In the study by Germain *et al.* [32], the metabolism of DADS, a decomposition product of allicin, was investigated in rats. The authors administered 200 mg/kg DADS to rats, and the metabolites present in stomach, liver, plasma and urine were monitored over a period of 15 days. DADS and allyl mercaptan in plasma were only detected at 20 min after oral administration of DADS, whereas the other metabolites, namely AMS, AMSO and AMSO_2_, were detectable at this time until 7 days after oral administration, which is in good agreement with the results of the present study. DADS and allyl mercaptan were not detectable at all in urine and the only metabolites were AMS, AMSO and AMSO_2_. Accordingly, it is possible that no allyl mercaptan is excreted into human milk, but this has to be confirmed in further experiments. In view of temporal profiles, Germain *et al.* [32] reported that all metabolites (allyl mercaptan, AMS, AMSO and AMSO_2_) were most abundant 48 to 72 h after DADS administration. In our study, we only monitored up to 5.2 h after garlic consumption; during this time it appears as if we already observed a maximum of some of the metabolites. However, in our human study, continued sampling was limited due to constraints regarding the natural breastfeeding regimes. Moreover, the rationale for the sampling period used in our study was that excretion maxima in humans for odorants and their metabolites have been reported to occur commonly around two to three hours after intake [7,13,14,15,48]. Regarding the administered concentrations, it has also to be mentioned that Germain *et al.* [32] applied 200 mg/kg of pure DADS to the rats whereas the test persons in our study only consumed 3 g of raw garlic. The allicin content of raw garlic lies between 0.35% and 0.53% [49,50]; under the assumption that the total amount of allicin is converted to DADS, 3 g of garlic accordingly relate to about 120 mg DADS (calculated with an allicin content of 0.4%). If one considers a human weight of about 60 kg, this amount represents just a fraction of the amount administered in the study by Germain *et al.* [32]. Accordingly, the experimental conditions are not directly comparable. Moreover, other garlic constituents might also influence the metabolic fate of the substances, and it is further possible that humans metabolize garlic differently, *i.e.*, faster than rodents, as garlic is a common part of their diet.

Apart from the *in vivo* study by Germain *et al.* [32], other authors studied the metabolism of garlic constituents based on rat tissues, cells or body fluids. Brady *et al.* [46] utilized liver, blood and urine samples from rats which were treated with 200 mg/kg DAS. In their studies they detected DASO as well as DASO_2_ in each of the samples. Egen-Schwind *et al.* [51,52] identified DADS and allyl mercaptan as metabolites of allicin in isolated perfused rat liver, and reported DADS as a precursor for allyl mercaptan. Sheen *et al.* [53] detected allyl mercaptan and AMS as metabolites of DADS in primary rat hepatocytes when treating the cells with 1 mM DADS or DAS dissolved in propylene glycol for different periods. DADS was almost completely converted to allyl mercaptan and AMS within 120 min. DAS, on the other hand, only led to the formation of AMS. This observation is in contrast to the results of Brady *et al.* [46], and contradicts the assumption of Lawson and Wang [26] who proposed DASO and DASO_2_ as metabolites of DAS.

In our study, neither allyl mercaptan nor DADS were detected, which is not in line with the findings of Egen-Schwind *et al.* [51,52] and Germain *et al.* [32]. An explanation for this observation might be that DADS and allyl mercaptan are rapidly converted to other metabolites, as proposed by Lawson and Wang [26], or that both substances are not excreted into human milk. On the other hand it is possible that allyl mercaptan is not detectable by the method used within this study. Allyl mercaptan is a very volatile compound and could be lost during the work-up procedure of the human milk samples. This aspect should be addressed in future quantification studies, ideally involving stable isotope dilution assays [8,11,12,54].

DADS is possibly completely converted to other metabolites in the human body, leading to the formation of compounds such as allyl mercaptan, AMS, AMSO and AMSO_2_, which could be the reason why it was not detected in our study. Likewise, DASO and DASO_2_ were not detected in our study, indicating that DAS might be metabolized differently in humans than in rats. However, it might also be that not all substances are transferred from blood to human milk; their presence in blood would also need to be investigated in further studies. On the other hand, there are studies reporting glutathione conjugates of DAS and its putative metabolites DASO and DASO_2_ [47], as well as *N*-acetyl conjugates. In view of this, it is important to note that De Rooij *et al.* [55], as well as Jandke and Spiteller [56], identified *N*-acetyl-*S*-allyl-l-cysteine in human urine after garlic consumption. This metabolite may be formed from the water-soluble *S*-allyl cysteine catalyzed by *N*-acetyl transferase. Even if excretion of these conjugates occurs via urine, the potential presence of such conjugates or less volatile metabolites in human milk would need to be regarded in future studies involving other techniques such as HPLC.

To conclude our findings, this study clearly shows that garlic consumption leads to a distinct sensory impact on the human milk aroma profile, even eliciting odor impressions that are related to the original aroma profile of the ingested food. At first sight, this might lead to the assumption that aroma transmission into milk occurs with a direct representation of the odorous substances of the garlic. Nevertheless, our study clearly shows that this sensory impression is misleading, and that other derivatives are found in the milk, whereby some are even odorless and only one bears the characteristic garlic smell, namely AMS. Accordingly, this study clearly demonstrates that the odor composition after transmission into human milk does not necessarily coincide with what is found in the original food, and that metabolism and other transmission or resorption processes may play a major role in human milk odor formation. This is in agreement with other studies of our group where it was either shown that nutritional odorants did not translate into human milk [7,8], or that substances were heavily metabolized and showed up as their derivatives [14]. More studies will be required in the future to elucidate which odorants or substance classes, or their respective precursors, bear the potential of influencing the odor profiles of human milk, and at which concentration levels. Only then will it be possible to gauge if babies can be influenced by such substance transmission, and if such influence would be only with sensory impact or if there is even the potential of other physiological effects. Especially metabolites are worth considering with regard to these aspects.

## 4. Materials and Methods

### 4.1. Chemicals/Materials

The following reference compounds were obtained from the suppliers shown: dimethyl disulfide (DMDS), dimethyl trisulfide (DMTS) (Sigma-Aldrich, Steinheim, Germany), allyl methyl disulfide (AMDS) (abcr, Karlsruhe, Germany). Allyl methyl sulfide (AMS), allyl methyl sulfone (AMSO_2_), allyl methyl sulfoxide (AMSO), diallyl disulfide (DADS), diallyl sulfide (DAS), diallyl sulfone (DASO_2_), diallyl sulfoxide (DASO), 3-vinyl-4*H*-1,2-dithiin and 2-vinyl-4*H*-1,3-dithiin, and diallyl trisulfide (DATS) were synthesized as described below.

Chemicals used for the syntheses of reference substances were obtained from the suppliers shown: bis(tri-*n*-butyltin)sulfide ((Bu_3_Sn)_2_S), Triton X 100 (abcr, Karlsruhe, Germany); acrolein diethyl acetal 96%, allyl bromide 97%, meta-chloroperoxybenzoic acid ≥ 99%, cobalt-(II)-chloride (CoCl_2_) 97%, 4,4-cyclohexylidenebis[*N*,*N*-bis(4-methylphenyl)benzenamine] (TAPC) 97%, ethanol absolute, hydrogen peroxide 30 wt % in H_2_O, magnesium sulfate (MgSO_4_) anhydrous, manganese-(IV)-oxide (MnO_2_) ≥ 99%, methyl iodide ≥ 99%, oxalic acid 98%, petroleum ether, polyethylene glycol 300 (PEG 300), 2-propen-1-thiol ~60%, pyridine anhydrous 99.8%, sodium bicarbonate (NaHCO_3_), sodium bisulfite (NaHSO_3_) solution ~40%, sodium carbonate (Na_2_CO_3_) anhydrous, sodium iodate (NaIO_3_) ≥ 99%, sodium thiosulfate pentahydrate ≥ 99.5%, tetrahydrofuran anhydrous, thiourea ≥ 99%, toluene anhydrous 99.8%, trimethylaluminum solution (2.0 M in toluene) (Sigma-Aldrich, Steinheim, Germany); dichloromethane (DCM) high performance liquid chromatography (HPLC) grade, ethyl acetate (EtOAc), hexane, silica gel 60 (SiO_2_), sodium hydroxide (NaOH), sodium sulfate anhydrous (Na_2_SO_4_), sodium sulfide anhydrous (Na_2_S) (VWR, Darmstadt, Germany). The solvents were freshly distilled prior to analysis.

### 4.2. Syntheses of Reference Substances

AMS was obtained from 2-propen-1-thiol by using the method described by [57] for the synthesis of unsymmetrical sulfides via a CoCl_2_ catalyzed reaction of thiols with allyl iodide under visible irradiation in the presence of pyridine. This was achieved by mixing 2-propen-1-thiol with methyl iodide, pyridine and CoCl_2_ and irradiating the mixture with a 500 W lamp for 15 min. After the mixture had cooled down to room temperature, the solution was extracted with diethyl ether. The deep red organic phase was separated, washed with water, dried over MgSO_4_, and the solvent evaporated under reduced pressure. The compound was further purified via column chromatography using hexane as solvent and SiO_2_ as stationary phase. Yield: 63 mg, 15.5 mmol, 39.65%. ^1^H-NMR (600 MHz, CDCl_3_): δH = 5.78 (1H, m, 2), 5.12 (1H, m, HC=CHH trans, 1), 5.09 (1H, m, HC=CHH cis, 1), 3.12 (2H, d, 3), 2.03 (3H, s, 4). ^13^C-NMR (150 MHz, CDCl_3_): δC = 134.23 (2), 117.08 (1), 33.30 (3), 14.05 (4). MS-EI: *m*/*z* (%) = 89 (5), 88 (100), 73 (69), 72 (6), 71 (6), 61 (15), 47 (13), 46 (12), 45 (34), 41 (26), 39 (25).

AMS was then further oxidized to AMSO as described by Oae, *et al.* [58]. The oxidizing agent MCPBA was used for the reaction with the difference that the powdered MCPBA was not added directly to the reaction mixture as described in the literature, but first dissolved in DCM and then added dropwise. The reaction mixture was then allowed to stir for 30 min instead of 4.5 h. An additional drying step with Na_2_SO_4_ was applied after neutralizing with NaHCO_3_ and washing with water. To obtain the crude product, the solvent was removed under reduced pressure at 30 °C. Further purification of the product was then accomplished by column chromatography with EtOAc. Yield: 0.206 g, 2.0 mmol, 70.64%. ^1^H-NMR (600 MHz, CDCl3): δH = 5.75 (1H, m, 2), 5.45 (1H, m, 1), 3.12 (2H, d, 3), 2.03 (3H, s, 4). ^13^C-NMR (150 MHz, CDCl_3_): δC = 125.52 (2), 128.81 (1), 53.41 (3), 14.21 (4). MS-EI: *m*/*z* (%) =104 (100), 103 (8), 89 (41), 87 (16), 76 (24), 75 (10), 73 (12), 72 (7), 71 (10), 61 (22), 59(14), 58 (17), 57 (50), 55(8), 49 (10), 48 (56), 46 (7), 45 (50), 41 (10).

Oxidation of AMS leads to the generation of AMSO_2_ when applying different conditions. The method described by Bland and Stammer [59] was used to achieve this, albeit with the reaction mixture left to stand for 4 days instead of 30 h. Yield: 0.421 g, 3.5 mmol, 89.44%. ^1^H-NMR (600 MHz, CDCl_3_): δH = 5.99 (1H, m, 2), 5.53 (1H, m, HC=CHH trans, 1), 5.49 (1H, m, HC=CHH cis, 1), 3.74 (2H, d, 3), 2.87 (3H, s, 4). ^13^C-NMR (600 MHz, CDCl3): δC = 125.40 (2), 124.69 (1), 59.53 (3), 39.05 (4). MS-EI: *m*/*z* (%) = 120 (19), 105 (5), 79 (7), 65 (6), 64 (10), 63 (8), 57 (6), 48 (5), 45 (6) 42 (7), 41 (100), 40 (8), 39 (86), 38 (10).

DADS was synthesized according to Firouzabadi, *et al.* [60]. However, the extraction and purification of the desired product was changed and performed as follows: Water was added and the mixture was subsequently extracted five times with EtOAc to extract the product from the reaction mixture. The combined organic phases were then dried over MgSO_4_ and the solvent was evaporated under reduced pressure. DADS was recovered as a colorless liquid after column chromatography over SiO_2_ with petroleum and evaporation of the solvent. Yield: 3.84 g, 26.25 mmol, 79%. ^1^H-NMR (600 MHz, CDCl_3_, RT): δ (ppm) = 5.86 (t, 2H, 2); 5.22–5.15 (dd, 4H, 1); 3.36 (t, 4H, 1). ^13^C-NMR (150 MHz, CDCl_3_, RT): δ (ppm) = 133.46 (2); 118.41 (1); 42.28 (3). MS-EI: *m*/*z* (%) = 146 (M^+^, 53), 113 (54), 105 (45), 103 (25), 85 (33), 81 (100), 79 (36), 73 (38), 71 (27), 45 (85).

Synthesis of DAS was carried out according to the method described by Lu and Cai [61], albeit using Trition X 100 rather than Trition X 10. Yield: 1.27 g, 11.12 mmol, 90%. ^1^H-NMR (600 MHz, CDCl_3_, RT): δ (ppm) = 5.76 (t, 2H, 2); 5.09–5.06 (dd, 4H, 1); 3.08 (t, 4H, 1). ^13^C-NMR (150 MHz, CDCl_3_, RT): δ (ppm) = 134.23 (2); 117.15 (1); 33.32 (3).MS-EI: *m*/*z* (%) = 114 (67), 99 (56), 81 (30), 80 (25), 73 (95), 72 (63), 71 (34), 45 (100), 41 (62), 39 (74).

The synthesis of DASO was carried out as described by Mokhtary *et al.* [62] with only minor changes applied: ethanol was used as solvent instead of methanol, and 20 mL of water was added for better separation of the phases. Finally, the extraction with DCM was performed twice instead of once. A mixture of hexane/ EtOAc (7/3) was used as solvent for the column chromatography. Yield: 3.87 g, 29.72 mmol, 83%. ^1^H-NMR (600 MHz, CDCl_3_, RT): δ (ppm) = 5.89 (m, 2H, 2); 5.41–5.50 (m, 4H, 1); 3.41–3.53 (m, 4H, 3). ^13^C-NMR (150 MHz, CDCl_3_, RT): δ (ppm) = 125.71 (2); 123.61 (1); 54.22 (3). MS-EI: *m*/*z* (%) = 100 (7), 82 (7), 81 (45), 80 (10), 79 (6), 73 (6), 68 (13), 67 (5), 45 (9), 41 (100).

DASO_2_ was additionally obtained from DAS. Synthesis was performed according to Bahrami, *et al.* [63]. Column chromatography over SiO_2_ with hexane/EtOAc (*v*/*v* 7/3) was applied for purification. Yield: 2.30 g, 15.73 mmol, 90%. ^1^H-NMR (600 MHz, CDCl_3_, RT): δ (ppm) = 5.83–5.95 (m, 2H, 2); 5.37–5.49 (dd, 4H, 1); 3.66–3.68 (d, 4H, 3). ^13^C-NMR (150 MHz, CDCl_3_, RT): δ (ppm) = 124.91 (2); 124.65 (1); 55.93 (3). MS-EI: *m*/*z* (%) = 81 (8), 67 (40), 54 (17), 41 (100).

3-Vinyl-4*H*-1,2-dithiiin was synthesized according to Li, *et al.* [64], albeit with a nitrogen instead of an argon atmosphere. Further deviations from the previously reported protocol were carried out after the addition of acrolein diethyl acetal: The reaction mixture was not poured into ice-water but stirred overnight at room temperature. The mixture was then poured into water and the organic layer was separated and treated as described by Li *et al*. [64] but with a hexane/DCM ratio of 4/1 (*v*/*v*) instead of 5/1 (*v*/*v*) for column chromatography. Yield: 0.021 g, 0.15 mmol, 5%. ^1^H NMR (600 MHz, CDCl_3_, RT): δ (ppm) = 5.95–6.08 (m, 2H, 3/4/5); 5.27–5.31 (m, 2H, 6); 4.71–3.75 (m, 1H, 1); 2.45–2.63 (m, 2H, 2). ^13^C NMR (150 MHz, CDCl_3_, RT): δ (ppm) = 135.90 (4); 125.47 (3); 120.06 (5); 117.01 (6); 43.60 (1); 30.41 (2). MS-EI: *m*/*z* (%) = 144 (91), 111 (100), 103 (55), 97 (62), 79 (34), 77 (40), 72 (33), 71 (41), 45 (30), 39 (21).

2-Vinyl-4*H*-1,3-dithiin was synthesized using the same procedure as for 3-vinyl-4*H*-1,2-dithiin. Yield: 0.22 g, 1.53 mmol, 47%. ^1^H-NMR (600 MHz, CDCl_3_, RT): δ (ppm) = 6.31 (d, 1H, 4); 5.99 (m, 2H, 3/5); 5.27–5.42 (dt, 2H, 6); 4.74 (d, 1H, 1); 3.27-3.40 (dd, 2H, 2). ^13^C-NMR (150 MHz, CDCl_3_, RT): δ (ppm) = 134.13 (4); 122.07 (3); 118.45 (5); 117.17 (6); 45.16 (1); 25.12 (2). MS-EI: *m*/*z* (%) = 144 (M^+^, 99), 111 (77), 103 (22), 97 (28), 85 (10), 79 (13), 73 (14), 72 (99), 71 (100), 45 (39).

The synthesis of DATS was performed according to the method described by Ren, *et al.* [65]. Small modifications on the synthesis protocol were made: After separation of the two layers, the aqueous layer was washed twice with DCM. The combined organic layers were washed with H_2_O and dried over MgSO_4_. After evaporation of the solvent the product was recovered as colorless liquid. No further column chromatography was performed as the substance had already the desired purity of 89%. Yield: 2.61 g, 14.64 mmol, 89%. ^1^H-NMR (600 MHz, CDCl_3_, RT): δ (ppm) = 5.89 (m, 2H, 2); 5.26 (t, 4H, 1); 3.52 (d, 4H, 3). ^13^C-NMR (150 MHz, CDCl_3_, RT): δ (ppm) = 132.70 (2); 119.09 (1); 41.67 (3). MS-EI: *m*/*z* (%) = 178 (M^+^, 8), 114 (10), 113 (100), 74 (7), 73 (91), 72 (9), 71 (10), 47 (7), 45 (30), 41 (53).

### 4.3. Human Milk Samples

Human milk samples were obtained from six volunteer donors. The volunteers (age range 25–40 years (mean 31)) had no known illnesses at the time of examination, their breast milk production was normal, and they produced milk in excess of their infants’ needs. The sampling took place in the lactation period from 22 to 51 (mean 30) weeks postpartum using a mechanical breast pump (Medela Harmony™, Medela AG, Baar, Switzerland). To reduce the amount of sulfurous substances in the human milk on the sampling day, each subject was asked not to eat food containing high amounts of sulfur compounds for two days prior to the intervention and also on the sampling day; foods to be avoided were garlic, onion, wild garlic, chives, cabbage and leek. Furthermore, mothers were instructed to keep a record of all foods and beverages they consumed during this three-day period. The respective dietary records can be found in the online supplementary material Tables S1–S6.

On the sampling day, donors were asked to ingest approx. 3 g raw garlic which was obtained from a local supermarket (Aldi-Süd, Erlangen, Germany and Aldi-Süd, Freising, Germany) and peeled and cut into approx. 3 mm cubes by using a garlic cutter (Genius GmbH, Germany). Three human milk samples (9.5 to 50 g) were provided by each mother: one prior to garlic consumption and two afterwards according to the normal lactation period of each woman (usually with two to three-hour intervals between sampling). Minor deviations in sampling time were accepted, to minimize disruption to the nursing intervals. The milk samples obtained were evaluated and analyzed immediately. A table with the exact sampling time and the amount of the milk samples is given in the online supplementary material Table S7.

### 4.4. Study Design

The study was conducted in agreement with the Declaration of Helsinki. Written consent was provided by all four volunteers before sampling and analysis after a full explanation of the nature and purpose of the study. Resignation from the study was possible at any time. The study (registration number 49_13B) was approved by the Ethical Committee of the Medical Faculty, Friedrich-Alexander-Universität Erlangen-Nürnberg.

### 4.5. Aroma Profile Analysis

The sensory analyses of the human milk samples were performed by trained volunteers from the University of Erlangen-Nürnberg (Erlangen, Germany) who exhibited no known illness at the time of examination and with audited olfactory and gustatory function. Panelists were trained at recognizing about 90 selected odorants and different odorant concentrations according to their odor qualities, and in naming these according to an in-house developed flavor language in weekly training sessions over at least four months prior to performing the sensory analyses of this study.

Orthonasal evaluation (smelling) of the human milk samples was performed by presenting the samples to the sensory panel in a brown glass bottle (capacity 50 mL) in a sensory room at 21 ± 1 °C. No information about the purpose of the experiment was given. The panelists were asked to score the intensities of different sensory attributes on a scale from 0 (no perception) to 3 (strong perception). Attributes used were as follows and were the same as already established for the odor of human milk by Sandgruber *et al.* [39]: hay-like, fishy, fatty, rancid, sweaty, metallic, grassy-green, sweet, egg white-like, and buttery; only the attribute lactic was added, as required by our sensory panel. Panelists were additionally asked to score the intensities of the two attributes garlic- and cabbage-like.

### 4.6. Solvent-Assisted Flavor Evaporation (SAFE) of Volatiles from Human Milk

Solvent-assisted flavor evaporation (SAFE) [66] was used for the isolation of the volatile compounds from the breast milk samples. DCM was added at a ratio of 1:2 (*v*/*v*) to 12–50 g milk samples. The solution was then stirred for 30 min and thereafter immediately applied for SAFE distillation at 50 °C. The distillate obtained was then extracted three times with 25 mL DCM and the combined DCM phases were dried over anhydrous Na_2_SO_4_ and concentrated to a total volume of 100 µL at 50 °C by means of Vigreux distillation and micro-distillation [67]. The extracts were stored at −20 °C until analysis. A blank sample comprised of 25 mL DCM that was worked up instead of a milk sample.

### 4.7. High-Resolution Gas Chromatography-Olfactometry (HRGC-O)

High-resolution gas chromatography−olfactometry (HRGC-O) was performed with a Trace Ultra GC (Thermo Finnigan, Dreieich, Germany) using the following capillaries: DB-FFAP (30 m × 0.32 mm, film thickness 0.25 µm; J&W Scientific, Fisons Instruments, Mainz-Kastel, Germany) and DB-5 (30 m × 0.32 mm, film thickness 0.25 µm; J&W Scientific).The effluent was split at the end of the capillaries between a sniffing port and a flame ionization detector (FID) using two deactivated, uncoated fused silica capillaries (i.d. 0.32 mm). The FID and the sniffing port were held at 250 °C and 270 °C, respectively. The flow rate of helium carrier gas was 2.0 mL/min. Administration of the samples was performed by the cold on-column technique, whereby 2.0 µL of the extract were injected manually into a cold-on-column injector at 40 °C directly on a pre-column of uncoated, deactivated fused silica capillary (2–3 m × 0.32 mm). The pre-column was changed regularly to avoid accumulation of contaminants.

### 4.8. Determination of Odor Qualities of Reference Compounds

HRGC-O was applied for determining the odor qualities of reference compounds according to the following instrument parameters: The helium carrier gas flow rate was set to 2.5 mL/min. The temperature program of the oven started at 40 °C and was raised to 240 °C (DB-FFAP) or 300 °C (DB-5) at a rate of 8 °C/min. DB-5 and DB-FFAP columns were used for DASO and DASO_2_, and AMSO and AMSO_2_, respectively. The reference compounds were diluted in DCM at the following concentrations: The concentration of AMSO and AMSO_2_ were 47.1 µg/mL and 49.0 µg/mL, respectively. The concentration of DASO was 50.2 µg/mL. Concentrations of 47.8 µg/mL and 477.5 µg/mL were applied for DASO_2_. The reference solutions comprised 2 µL, which were injected manually. The odor qualities of all substances were determined by 10–12 panelists by sniffing the effluent at the sniffing port.

### 4.9. Comparative Aroma Extract Dilution Analysis (cAEDA)

Comparative aroma extract dilution analysis (cAEDA) was used to determine the flavor dilution (FD) factors of the odor compounds in human milk before and after garlic consumption [40,41]. The original extracts of 100 µL were thereby diluted stepwise (1 + 1, *v*/*v*) in DCM. HRGC-O was then performed on 2 µL of the original extracts (FD = 1) and the respective dilutions on a DB-5 column. The temperature program for the GC oven was as follows: After holding an initial temperature of 40 °C for 7 min the temperature of the GC oven was raised to 250 °C at 8 °C/min and then held for 5 min. The odorants were screened by two panelists who sniffed the effluent after gas chromatographic separation. Linear retention indices (RIs) of the compounds were calculated as described by Van den Dool and Kratz [68].

### 4.10. High-Resolution Gas Chromatography−Mass Spectrometry (HRGC-MS)

The characteristic mass spectra (EI, CI) of eluting compounds were obtained on an Agilent MSD quadrupole system (GC 7890A and MSD 5975C, Agilent Technologies, Waldbronn, Germany) equipped with a GERSTEL CIS 4 injection system and GERSTEL MPS 2 autosampler (GERSTEL, Duisburg, Germany). The software used to record the mass spectra and perform the data analysis was MSD ChemStation E.02.00.493 (Agilent Technologies). DB-FFAP and DB-5 (30 m × 0.25 mm, film thickness 0.25 µm, Agilent J&W Scientific, Santa Clara, USA) were used in the GC. An uncoated, deactivated fused silica capillary was used as a pre-column (2–3 m × 0.53 mm) and changed regularly to avoid accumulation of impurities. Helium was used as carrier gas and the total flow of the system was 1.0 mL/min, which was transferred into the MS using an uncoated, deactivated fused silica capillary (0.3–0.6 m × 0.25 mm) transfer line. EI mass spectra were generated in full scan mode (*m*/*z* 30–350) at 70 eV. The GC oven was held at 40 °C for 7 min, then raised to 240 °C and 250 °C for FFAP and DB-5, respectively, at a rate of 8 °C/min and held for 7 min.

### 4.11. Two-Dimensional High-Resolution Gas Chromatography−Mass Spectrometry/Olfactometry (HRGC-GC-MS/O) (Heart-Cut)

A two-dimensional gas chromatographic system was used for mass spectrometric identification of trace constituents. It consisted of two Varian 450 GCs in combination with a Varian 220 MS ion trap mass spectrometer (Varian, Darmstadt, Germany). The first GC was equipped with a GERSTEL MCS 2 multi-column switching system and was connected to the second GC by a GERSTEL CTS 1 cryo-trap system (GERSTEL, Duisburg, Germany). A DB-FFAP column (30 m × 0.32 mm, film thickness 0.25 mm (Agilent J&W Scientific, Santa Clara, CA, USA); first oven) and a Rxi-5HT column (30 m × 0.25 mm, film thickness 0.25 mm (Restek, Bad Homburg, Germany); second oven) were used. An uncoated, deactivated fused silica capillary was used as pre-column (2–3 m × 0.53 mm). The flow rate of helium carrier gas was 2.5 mL/min. The effluent was split in the first oven between an olfactory detection port (ODP, GERSTEL) and an FID, as well as a cryo-trap during the cut interval. In the second oven, the effluent was split toward a second ODP and the mass spectrometer. All split capillaries were made of uncoated, deactivated fused silica material. The FID and the sniffing ports were held at 250 and 260 °C, respectively. Mass spectra (*m*/*z* 35–300) in EI mode were generated at 70 eV ionization energy. The cut time intervals on the main column were determined by injection of the respective reference substances. Application of the samples to the GC system was performed at 40 °C using the cold-on-column technique. The temperature programs were as follows: For AMS: The first oven at 40 °C was held for 7 min and then raised to 240 °C at a rate of 8 °C/min, then kept for 5 min. The second oven started at a temperature of 40 °C, was held for 7 min and then raised to 250 °C at a rate of 8 °C/min. Afterwards, it was further raised to 300 °C at a rate of 25 °C/min and held for 5 min. For AMSO/AMSO_2_: The first oven started at a temperature of 40 °C, which was then raised to 150 °C at a rate of 20 °C/min and further to 240 °C at a rate of 8 °C/min. The oven at 240 °C was then held for 5 min. The second oven started at a temperature of 40 °C and was raised to 250 °C at a rate of 8 °C/min. For DAS, DATS, DMTS, DMDS, AMDS, DADS; 2-vinyl-4*H*-1,3-dithiin and 3-vinyl-4*H*-1,2-dithiin: The first oven was heated as described above for AMSO/AMSO_2_. The second oven started at a temperature of 40 °C, which was held for 2 min. It was then raised to 250 °C at a rate of 8 °C/min.

### 4.12. Identification of Metabolites and Calculation of Metabolite Profiles

Metabolites of garlic constituents in human milk were identified by comparing retention indices according to Van den Dool and Kratz [68]. Their odors were subsequently perceived at the sniffing port via GC-O, and by comparison of EI mass spectra generated by either HRGC-MS or HRGC-GC-MS/O with those of synthesized references. Comparison of the mass spectra of the analyte with the reference standard was performed using the NIST Mass Spectral Search Program (Version 2.0 d, National Institute of Standards and Technology, Gaithersburg, MD, USA). The identification was ranked positive if reverse match values were above 900. For calculation of retention indices, two analytical capillaries of different polarities were used (DB-FFAP and DB-5).

The time dependency of metabolite formation and excretion, specifically in relation to potential inter-individual differences, was assessed based on the relative concentration of the metabolites in different human milk samples, as follows: AMS was determined by 2D-HRGC-MS, with *m*/*z* 73 and 88 extracted from the total ion chromatogram and the area of the resulting peak then determined. The peak area was then normalized to the amount of the human milk (in kg) in order to express the concentration in units of area/kg milk. AMSO and AMSO_2_ were determined by HRGC-MS, with *m*/*z* 104 and 120 extracted, respectively. The peak areas were determined and the concentrations were calculated as described for AMS.

## 5. Conclusions

Several novel aspects could be successfully demonstrated in this study: First, sensory evaluation by an expert panel showed that the intake of a dietary-relevant amount of raw garlic changed the odor of breast milk, leading to garlic/cabbage-like odor notes being detectable in the milk. Accordingly, the expert panel confirmed previous observations made by naïve mothers and further specified the nature of the sensory changes observed. Second, garlic-derived metabolites were successfully identified in the milk by chemo-analytical means for the first time. These were allyl methyl disulfide (AMS), allyl methyl sulfoxide (AMSO) and allyl methyl sulfone (AMSO_2)_, whereby AMS was found to be the only odor-active substance eliciting a garlic-like odor. It is important to note that despite its garlic odor attribute, the substance present in the milk does not chemically represent the characteristic odorant profile of garlic itself [27,28,29,30,31,35,36,37,38]. Accordingly, the transmission of an odorous substance into human milk does not necessarily reflect the chemical composition of the original aroma in the food consumed, as has been commonly proposed in previous studies.

The metabolites detected were monitored for up to 5.2 h after garlic consumption. It could thereby be shown that metabolite profiles differed between individuals, resulting in different temporal profiles and the occurrence of different maximum concentration levels of the substances monitored. AMSO and AMSO_2_ are thus assumed to be oxidation products of AMS. Diallyl sulfoxide (DASO) and diallyl sulfone (DASO_2_), which were assumed to be oxidation products of diallyl sulfide (DAS), were not detectable. Future studies are needed to elucidate the potential occurrence of other less volatile metabolites in breast milk. Likewise, the potential physiological impact of garlic metabolites in human milk on the infant should be addressed in future studies.

## Figures and Tables

**Table 1:** Retention indices on DB-FFAP and DB-5 chromatographic capillaries and odor qualities of all substances investigated in this study. Literature reports on these substances in human or animal studies are provided together with an indication of their presence in breast milk samples in this study.

Substance (Abbreviation)	Retention Index (RI)		Identified in Milk after Garlic Intake ^a^	Odor Quality	Previously Detected in/Described as	Reference
	FFAP	DB-5				
Allyl methyl sulfide (AMS)	<1000	715	+ ^b^	garlic-like ^c,d^	Human breath after garlic consumption	[17,18,19,20,21,22,23,24,25,26]
					Human urine after garlic consumption	[24]
					Garlic	[27,28,29,30,31]
Allyl methyl sufloxide (AMSO)	1742	1018	+	odorless ^e^	Identified in rat stomach, liver, plasma and urine after administration of diallyl disulfide (DADS)	[32]
					Garlic metabolite in the human body	
Allyl methyl sulfone (AMSO_2_)	1983	1061	+	odorless ^e^	Identified in rat stomach, liver, plasma and urine after administration of DADS	[26,32]
					Garlic metabolite in the human body	
Diallyl sulfoxide (DASO)	1889	1163	-	garlic-like ^e^	Potential garlic metabolite in the human body	
Diallyl sulfone (DASO_2_)	2079	1289	-	odorless ^e^	Potential garlic metabolite in the human body	[26]
Diallyl disulfide (DADS)	1462	1083	-	garlic-like ^c,d^	Human breath after garlic consumption	[17,19,20,21,22,23,24,25,33,34]
				pungent ^d^	Garlic	[28,29,30,35,36,37]
Allyl methyl disulfide (AMDS)	1265	921	-	garlic-like, cooked garlic-like ^d^	Human breath after garlic consumption	[18,19,20,24,25]
					Garlic	[27,28,29,30,31,38]
Dimethyl disulfide (DMDS)	1071	751	-	cabbage-like ^c^ cooked garlic-like, onion-like, rubber-like ^d^	Human breath after garlic consumption	[18]
					Garlic	[27,28,29,30,31,36]
Dimethyl trisulfide (DMTS)	1362	973	-	garlic-like ^c^	Garlic	[27,29,30,31,36]
				burnt garlic-like, diffusive, penetrating, sulfury ^d^		
Diallyl trisulfide (DATS)	1771	1308	-	garlic-like ^c^	Human breath after garlic consumption	[18,25]
				garlic-like, onion-like ^d^	Garlic	[27,28,29,30,31,35,36,37,38]
Diallyl sulfide (DAS)	1138	868	-	garlic-like ^c^	Human breath after garlic consumption	[18,21,22,23,25]
					Garlic	[27,28,29,30,31,35,36,37]
2-Vinyl-4*H*-1,3-dithiin	1827	1222	-	garlic-like ^c^	Garlic	[27,31,36,37,38]
3-Vinyl-4*H*-1,2-dithiin	1720	1194	-	Pungent garlic‑like ^c^	Garlic	[27,31,36,37,38]

^a^ + identified in the sample extracts via GC-GC-MS in comparison to the corresponding reference compound; not detected via GC-GC-MS in the sample extract, with relation to the respective reference substance; ^b^ detected additionally via GC-O; ^c^ odor quality of the substance as described in [29]; ^d^ odor quality of the substance as described in [28]; ^e^ odor determination was performed via GC-O using a reference solution.

## Supplementary Materials

The following are available online at www.mdpi.com/ 2218-1989/6/2/18/s1, Figure S1: Aroma profiles of human milk samples of test persons *a–f*. The samples were collected at different times before and after ingestion of 3 g of raw garlic, Tables S1–S6: Dietary records of test persons *a–f*, Table S7: Time of sampling, garlic consumption and the respective amounts of the gathered samples.

## Abbreviations

The following abbreviations are used in this manuscript:

AMDS	Allyl methyl disulfide
AMS	Allyl methyl sulfide
AMSO	Allyl methyl sulfoxide
AMSO_2_	Allyl methyl sulfone
APA	Aroma profile analysis
cAEDA	Comparative aroma extract dilution analysis
DADS	Diallyl disulfide
DAS	Diallyl sulfide
DASO	Diallyl sulfoxide
DASO_2_	Diallyl sulfone
DATS	Diallyl trisulfide
DMDS	Dimethyl disulfide
DMTS	Dimethyl trisulfide
FD-factor	Flavor dilution factor
FID	Flame ionization detector
HRGC-GC-MS/O	Two-dimensional high-resolution gas chromatography−mass spectrometry/olfactometry
HRGC-MS	High-resolution gas chromatography−mass spectrometry
RI	Linear retention indices
SAFE	Solvent-assisted flavor evaporation
