# Supplementary material for: Detection of Volatile Metabolites of Garlic in Human Breast Milk
Source: Metabolites. 2016 Jun 6;6(2):18. doi: 10.3390/metabo6020018 (PMC4931549; doi:10.3390/metabo6020018)
Supplement: Supplementary file 1 [file metabolites-06-00018-s001.pdf]

Supplementary Materials: Detection of Volatile Metabolites of Garlic in Human Breast Milk

Laura Scheffler, Yvonne Sauermann, Gina Zeh, Katharina Hauf, Anja Heinlein, Constanze Sharapa and Andrea Buettner

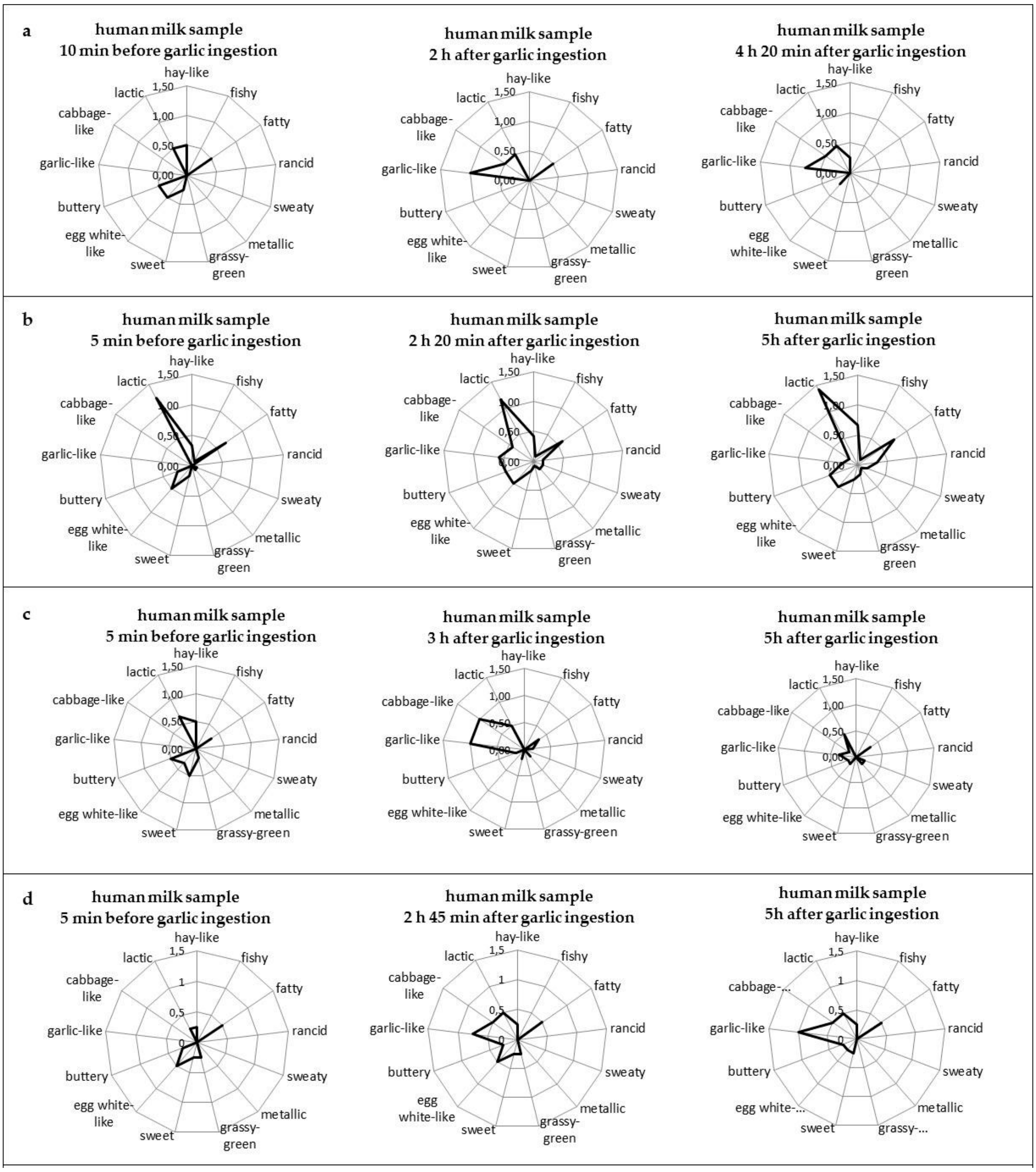

Figure S1. *Cont.*

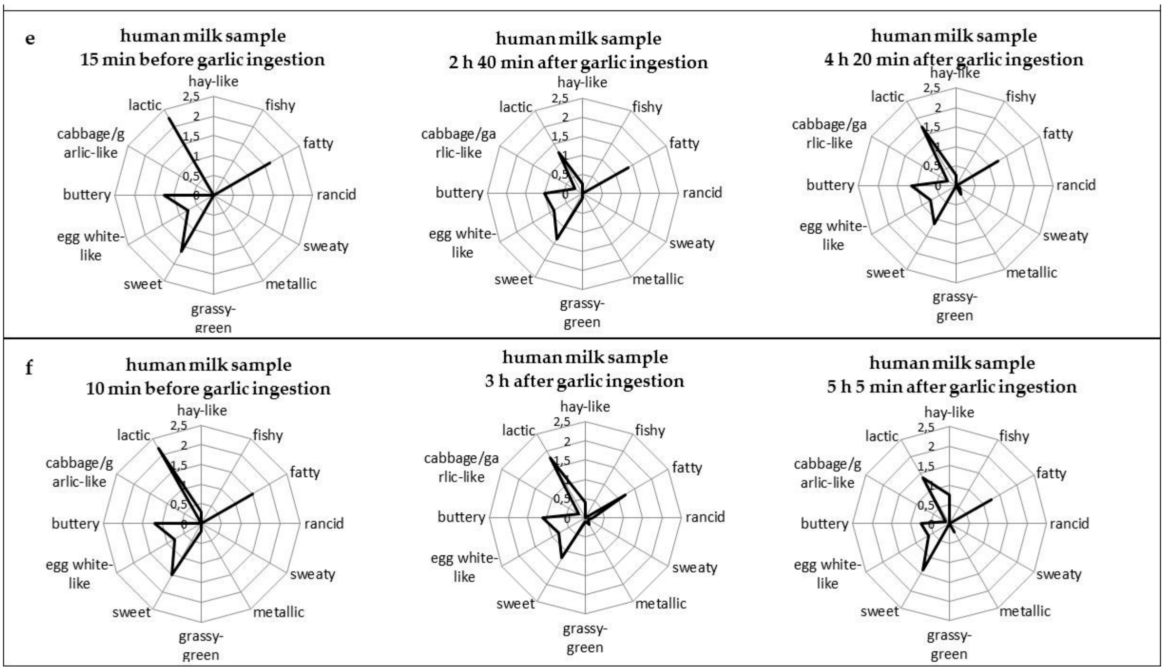

Figure S1. Aroma profiles of human milk samples of test persons (**a**–**f**).

Table S1. Dietary record-test person *a*.

Day of Dietary Record	Type of Meal	Food	Beverages
Day 1	break-fast	cereal	coffee
	S1	apple	fennel-anis-caraway-tea
	lunch	pasta with cheese sauce	coffee, water
	S2	apple, banana	
	dinner	bread with cheese	water
	S3		
Day 2	break-fast	cereal	coffee, fennel-anis-caraway-tea
	S1	apple	
	lunch	semolina porridge with berries	coffee, water
	S2	apple, chocolate cake	
	dinner	bread, pretzel with cheese, aspic	water
	S3		
Sampling Day	break-fast	croissant, apple, banana	
	S1		
	lunch		
	S2		
	dinner		
	S3		

S1, S2, S3: Snacks.

Table S2. Dietary record-test person *b*.

Day of Dietary Record	Type of Meal	Food	Beverages
Day 1	break-fast	cereal with fruits, amaranth and milk, α-Thyroxin 50 mg	cappuccino
	S1		water
	lunch	tofu-stir-fried vegetables with rice and curry sauce	water
	S2	chocolate croissant	cappuccino
	dinner	sugared pancake with raisins with apple sauce	water
	S3	½ banana	water
Day 2	break-fast	cereal with fruits, amaranth and milk, α-Thyroxin 50 mg	cappuccino
	S1	1 banana	water
	lunch	mozzarella-tomato with balsamic vinegar and pretzel	water
	S2	½ Bavarian doughnut	cappuccino
	dinner	pasta with soy Bolognese sauce and salad (cucumber, tomato, carrot, sunflower seeds, pumpkin seeds)	water
	S3		water
Sampling Day	break-fast	whole-grain bread with quark, currant-jelly, honey and butter	cacao
	S1		
	lunch	turkish spinach cake, endive salad with carrots	water
	S2		
	dinner		
	S3		

S1, S2, S3: Snacks.

Table S3. Dietary record-test person *c*.

Day of Dietary Record	Type of Meal	Food	Beverages
Day 1	break-fast	cheese roll	coffee
	S1		
	lunch	roll with chicken, salad and sauce (without garlic)	thyme tea, water
	S2		
	dinner	pasta with tomato sauce (mushrooms, tomatoes)	water, apple juice
	S3	chocolate	
Day 2	break-fast	cereal (grains, apple, milk)	coffee
	S1		
	lunch	cake with nuts, roll with herbs	thyme tea, water
	S2	rice pudding with cherries	coconut milk
	dinner	pasta with tomato sauce (mushrooms, tomatoes)	water
	S3	potato chips	
Sampling Day	break-fast	cereal (grains, apple, milk)	coffee, water
	S1		
	lunch	cheese roll	
	S2	chocolate	
	dinner		
	S3		

S1, S2, S3: Snacks.

Table S4. Dietary record-test person *d*.

Day of Dietary Record	Type of Meal	Food	Beverages
Day 1	break-fast	cereal with wild berries	blood orange juice
	S1		
	lunch	bread and ham	
	S2	chocolate	
	dinner	gratin	
	S3		nursing tea
Day 2	break-fast	cereal with wild berries	blood orange juice
	S1		
	lunch	gratin	
	S2		oriental, red tea
	dinner	gratin, mandarins	
	S3	cookies	good-night tea
Sampling Day	break-fast	cereal with wild berries	blood orange juice
	S1		
	lunch	gratin: zucchini, tomato, feta cheese, egg	
	S2	chocolate	
	dinner		
	S3		

S1, S2, S3: Snacks.

Table S5. Dietary record-test person *e*.

Day of Dietary Record	Type of Meal	Food	Beverages
Day 1	break-fast	3 slices rye-wheat bread, butter, Nutella, 2 apricots	1 tall cup of coffee with whole milk, 1 glass of ACE-spritzer
	S1		2 glasses of water
	lunch	polenta with stir-fried vegetables (zucchini, pepper, kohlrabi)	1 glass of water
	S2	carrot, nectarine and banana	2 glasses of water
	dinner	5 slices rye-wheat bread (1x with butter, 2x with liverwurst, 2x with cheese	1 glass of water
	S3		2 glasses of water, 1 glass of currant spritzer
Day 2	break-fast	3 slices rye-wheat bread, 1x liverwurst, 2x butter and marmalade, ½ apricot, ½ nectarine	1 tall cup of coffee with whole milk, 1 glass of currant spritzer
	S1		1 glass of water, 1 glass of currant spritzer
	lunch	3 slices rye-wheat bread with cheese, carrot	1 glass of water
	S2	nectarine, apricot, Hanuta	2 glasses of water
	dinner	whole-grain pasta with minced meat sauce (beef mince, tomato, pepper, peas), cheese	1 glass of water
	S3	Hanuta, 2 Kinder-Schoko-Bons	1 glass of water, 1 glass of tonic
Sampling Day	break-fast	3 slices rye-wheat bread with Nutella, 1 apricot, ½ nectarine	1 tall cup of coffee with whole milk, 2 glasses of water
	S1		2 glasses of water, 1 glass of apple spritzer
	lunch	whole-grain pasta with minced meat sauce (beef mince, tomato, pepper, peas), cheese	1 glass of water
	S2	Hanuta	1 small cup of coffee with milk, 1 glass of water
	dinner		
	S3		

S1, S2, S3: Snacks.

Table S6. Dietary record- test person *f*.

Day of Dietary Record	Type of Meal	Food	Beverages
Day 1	break-fast	3 slices rye-wheat bread	coffee with soy milk
	S1		
	lunch	noodles with olive-tomato sauce (tomato purée, olives, basil, provençal herbs, olive oil, pizza soft cheese)	
	S2	trail mix, gummi bears	
	dinner	hash browns with apple sauce	
	S3		
Day 2	break-fast	Alnatura breakfast puree with rice milk	nursing tea (fenugreek, fennel, anise, caraway, lemon verbena)
	S1		
	lunch	eggplants with tomato sauce (according to the restaurant without onions and garlic, but with broth from Maggi)	
	S2		
	dinner	bread with margarine and ham, cucumber	
	S3	dark chocolate	
Sampling Day	break-fast	vegan marble cake	coffee with soy milk, Fanta
	S1		
	lunch	bread bun with butter, ham, tomato, cucumber	water
	S2		water
	dinner		
	S3		

S1, S2, S3: Snacks.

Table S7. Time of sampling, garlic consumption and the respective amounts of the gathered samples.

Subject	Event	Time	Amount of Human Milk (g)
test person *a*	1st sample	9:00	437,953
	garlic consumption	9:10	
	2nd sample	11:10	118,489
	3rd sample	13:30	490,093
test person *b*	1st sample	9:20	337,798
	garlic consumption	9:25	
	2nd sample	11:45	277,028
	3rd sample	14:30	154,362
test person *c*	1st sample	9:05	159,959
	garlic consumption	09:10	
	2nd sample	12:10	185,397
	3rd sample	14:00	125,154
test person *d*	1st sample	9:20	152,956
	garlic consumption	9:25	
	2nd sample	12:10	131,801
	3rd sample	14:20	261,274
test person *e*	1st sample	9:40	436,317
	garlic consumption	9:55	
	2nd sample	12:35	446,195
	3rd sample	14:15	231,551
test person *f*	1st sample	9:35	326,611
	garlic consumption	9:45	
	2nd sample	12:45	95,967
	3rd sample	14:50	112,297
